# Transgenic FingRs for Live Mapping of Synaptic Dynamics in Genetically-Defined Neurons

**DOI:** 10.1038/srep18734

**Published:** 2016-01-05

**Authors:** Jong-Hyun Son, Matthew D. Keefe, Tamara J. Stevenson, Joshua P. Barrios, Scott Anjewierden, James B. Newton, Adam D. Douglass, Joshua L. Bonkowsky

**Affiliations:** 1 Department of Pediatrics, University of Utah School of Medicine, Salt Lake City, Utah; 2 Department of Neurobiology and Anatomy, University of Utah School of Medicine, Salt Lake City, Utah; 3 Interdepartmental Program in Neurosciences, University of Utah School of Medicine, Salt Lake City, Utah

## Abstract

Tools for genetically-determined visualization of synaptic circuits and interactions are necessary to build connectomics of the vertebrate brain and to screen synaptic properties in neurological disease models. Here we develop a transgenic FingR (fibronectin intrabodies generated by mRNA display) technology for monitoring synapses in live zebrafish. We demonstrate FingR labeling of defined excitatory and inhibitory synapses, and show FingR applicability for dissecting synapse dynamics in normal and disease states. Using our system we show that chronic hypoxia, associated with neurological defects in preterm birth, affects dopaminergic neuron synapse number depending on the developmental timing of hypoxia.

Vertebrate brain function depends on the structure and activity of complex circuits that are genetically defined but are shaped by development. Determining the identity, connections, and properties of the neurons that act at each step of a circuit can provide a foundation for understanding the basic principles underlying its function^1^. Despite recent successes in mapping vertebrate brain connectivity^2^,^3^,^4^ there is a paucity of genetic tools to visualize and manipulate neurons and their microstructural elements such as axons and synapses. Significant hurdles include the lack of reagents to visualize synapses in live animals; and that synapses from genetically distinct neurons cannot be differentiated using immunohistochemical methods. Further, screening for diseases affecting the synapse depends on reliable, fluorescent markers that can be used *in vivo*. While use of endogenous fluorophores tagged to synapse-localized proteins has been used successfully in transient analysis^5^,^6^, in stable transgenic lines the fluorescently-tagged synaptic proteins often fail to specifically localize (JLB, personal observation). Recently, a novel approach based on recombinant fibronectin intrabodies generated by mRNA display (FingRs) has been introduced for use in tissue culture and in mouse brain slices^7^. FingRs are antibody-like proteins that have been selected to target endogenous synaptic proteins including postsynaptic density 95 (PSD-95) and Gephyrin (GPHN). PSD-95 is a scaffolding protein localized to the postsynaptic density of excitatory synapses^8^, and GPHN is a component of the postsynaptic protein network of inhibitory synapses^9^. FingRs have several advantages over current methods: they are recombinant and can be expressed in cell types of interest; they can be fused to a fluorophore to permit visualization of synapses *in vivo*; and finally, they have an auto-feedback mechanism to limit overexpression that could affect synapse properties as well as obscure precise imaging. However, application of FingRs has not been demonstrated for use in an experimental analysis, and implementation in live animals with genetically-defined control of expression would expand the repertoire of potential uses.

## Results

We sought to adapt FingRs technology for use in a transgenic system in the small vertebrate zebrafish (*Danio rerio*). Embryonic and larval zebrafish are transparent, so that localization and dynamics of FingRs and synapses could be monitored in real-time *in vivo*. Further, with the Gal4/UAS system^10^ FingRs could be inducibly expressed under control of a UAS promoter to permit combinatorial expression in different neuron types and/or with different reporter types including for axons or for different synapse reporters.

**Figure 1:**
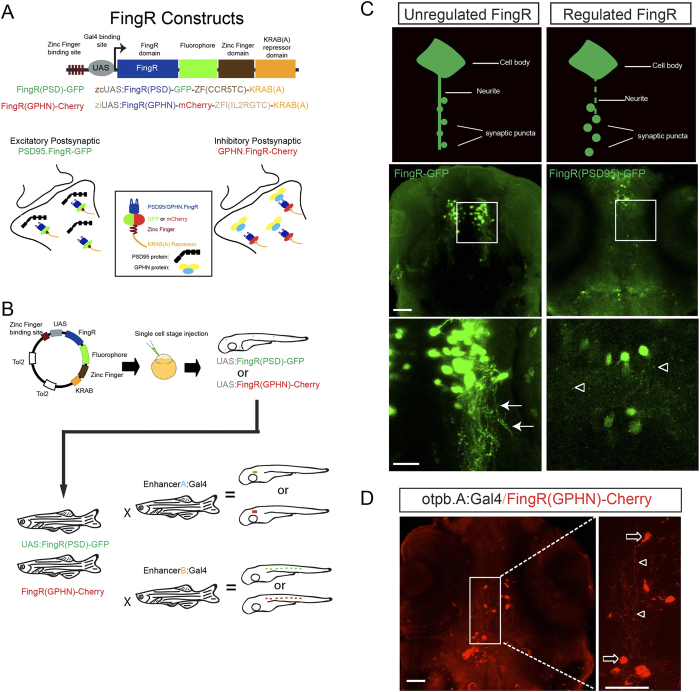
FingR constructs and testing in zebrafish. (**A**) Schematic diagram of FingR constructs used to generate plasmids and transgenic lines. FingR(PSD95)-GFP binds endogenous PSD-95 protein at the post-synaptic density; FingR(GPHN)-Cherry binds endogenous GPHN. (**B**) Use of inducible Gal4/UAS system with FingR(GPHN) or FingR(PSD95) in different Gal4 lines for differential synapse labeling (drawn by JHS). (**C**) Contrasting examples of unregulated (FingR-GFP) and regulated (FingR(PSD95)-GFP) FingR plasmids injected into Tg(*otpb.A.Gal4*) embryos. GFP signal from the unregulated FingR is distributed throughout the neuron and neurites (arrows) and individual synaptic puncta are not visualizable. In contrast, distinct puncta are seen (open arrowheads) using regulated FingR expression. Confocal images, scale bar 50 μm, 10 μm in inset panels; confocal z-stacks, ventral views, rostral to the top. D) Demonstration of puncta labeling with regulated FingR for GPHN. Confocal z-stacks, ventral view, rostral to top, of Tg(*otpb.A*:*Gal4*) embryo injected with FingR(GPHN)-Cherry. Arrows, neuron soma; arrowheads, puncta. Scale bar 50 μm, 10 μm in inset panel.

We cloned PSD95-FingR and GPHN-FingR into Tol2-based transposon UAS plasmids, and fused the FingRs to either a GFP or mCherry reporter (Fig. 1A,B). The domains used to generate the FingR constructs of PSD95 and GPHN share 75% and 86% similarity between mouse and zebrafish, suggesting a reasonable likelihood that the FingRs could be functional in zebrafish. Injections of UAS:PSD95.FingR-GFP plasmid into embryos of the line Tg(*otpb.A*:*Gal4*), which expresses in diencephalic dopaminergic neurons^11^, resulted in GFP expression in neuronal cell bodies and neurites (Fig. 1C). However, similar to the observation of Gross *et al.*, the high levels of FingR-GFP signal did not permit distinct synaptic puncta to be distinguished. To prevent over-expression of FingRs we modified our original unregulated UAS expression constructs to have a regulated transcriptional feedback mechanism using the previously described approach of Gross *et al.*^7^. We inserted the DNA binding site for a zinc-finger domain upstream of the UAS; and we modified the FingR constructs to include a zinc-finger DNA-binding domain, and a KRAB(A) transcriptional repressor. The final plasmid was composed of the zinc-finger DNA binding site, using distinct binding sites for PSD95.FingR and GPHN.FingR; the UAS binding site for Gal4; the FingR domain for PSD-95 or for GPHN; the fluorophore GFP or mCherry; the zinc-finger domain CCR5TC or IL2RGTC; and the KRAB(A) repressor domain (Fig. 1A,B). Gal4 drives expression of the FingR fusion protein, and the FingR will bind to PSD-95 or GPHN and the associated fluorescence will indicate the post-synaptic region. Once binding of the FingR to endogenous PSD-95 or GPHN is saturated, the excess FingR fusion proteins will bind to the zinc-finger sequence upstream of the UAS, and the KRAB(A) domain will inhibit further transcription. Using the regulated constructs, we found that plasmid injection of zcUAS:PSD95.FingR-GPF-ZFC(CCR5TC)-KRAB(A) (abbreviated FingR(PSD95)-GFP) or ziUAS:GPHN.FingR-mCherry-ZFI(IL2RGTC)-KRAB(A) (abbreviated FingR(GPHN)-Cherry) led to punctate labeling consistent with expression in post-synaptic regions (Fig. 1C,D).

**Figure 2:**
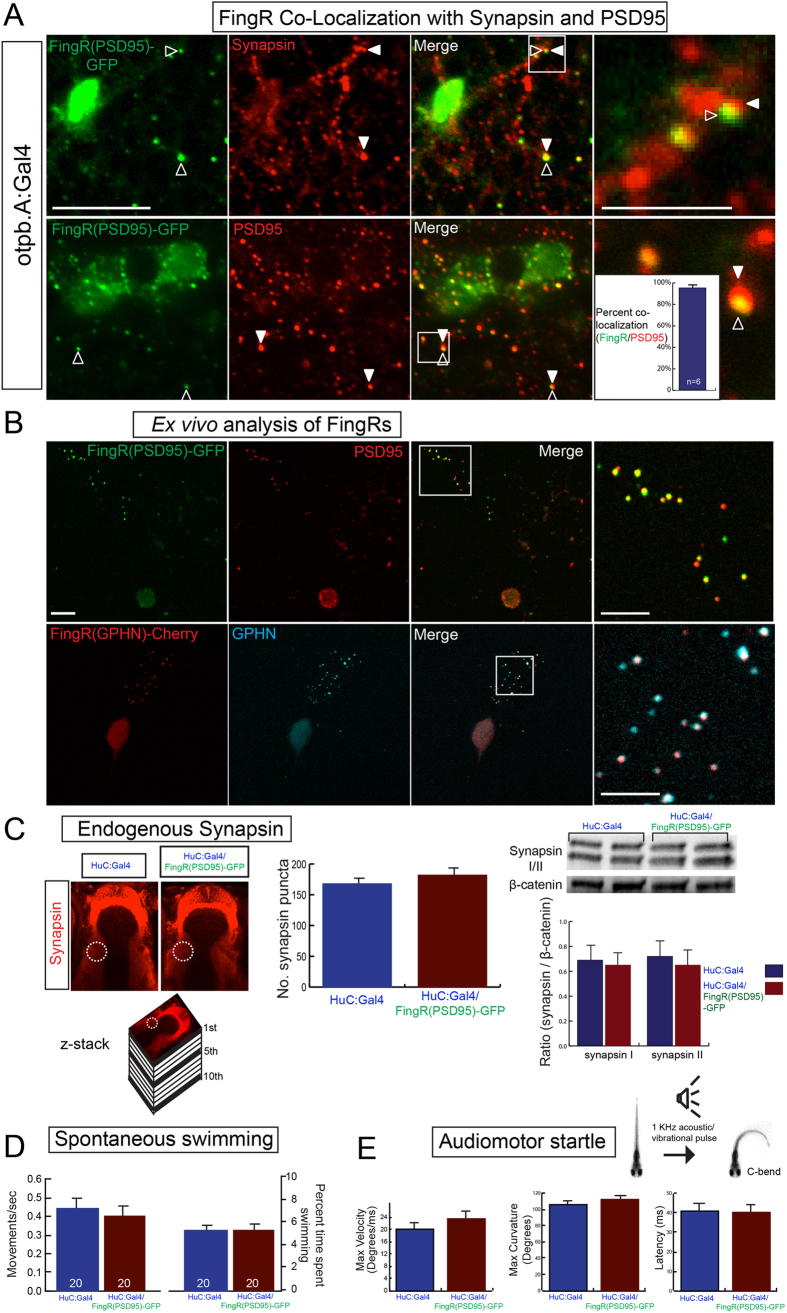
Transgenic FingR lines reflect endogenous synapses and do not affect synapse number or behavior. (**A**) FingR(PSD95)-GFP signal is adjacent to Synapsin immunohistochemistry (top panels) and overlaps PSD-95 immunohistochemistry (bottom panels). Quantification of FingR(PSD95)-GFP signal and endogenous PSD-95 signal demonstrates 95% overlap of PSD-95 immunohistochemistry with GFP signal from FingR (bar graph: median 95% +/− 1%, SEM; n = 6 larvae; *p* 0.01). Confocal images of sections from immunostained Tg(*otpb.A*:*Gal4*); Tg(*FingR*(*PSD95*)*-GFP*) larvae, scale bar 10 μm, 5 μm in inset panels. (**B**) *Ex vivo* sparse zebrafish primary neuron cell culture demonstrates co-localization of FingRs and endogenous synaptic proteins. Top row, dissociated neurons from Tg(*otpb.A*:*Gal4*); Tg(*FingR*(*PSD95*)*-GFP*) embryo, immunohistochemistry for anti-GFP and anti-PSD95. Bottom row from Tg(*HuC*:*Gal4*); Tg(*FingR*(*GPHN*)*-Cherry*) embryo, immunohistochemistry for anti-mCherry and anti-GPHN. Confocal images of slides, scale bar 10 μm, 5 μm in inset. (**C**–**E**) Pan-neuronal expression in Tg(*FingR*(*PSD95*)*-GFP*); Tg(*HuC*:*Gal4*) larvae. (**C**) FingR(PSD95)-GFP expression does not affect Synapsin protein expression. Data is double-transgenic larvae compared to Tg(*HuC*:*Gal4*) only. Images are confocal z-stacks, dorsal views, rostral to top; dotted circle indicates area of Synapsin puncta quantification for bar graphs. Western blots are for Synapsin in whole larvae, standardized to β-catenin; quantified bar graphs to right. (**D**) FingR(PSD95)-GFP expression does not affect spontaneous swimming behavior (percent time swimming and number of movements), n = 20 embryos each test; three separate experiments; SEM. (**E**) FingR(PSD95)-GFP expression does not affect audiomotor startle response, n = 27 (control) and 30 (FingR) embryos each test; three separate experiments, SEM (velocity, body curvature, or latency).

We generated stable transgenic lines carrying the FingR constructs Tg(*FingR*(*PSD95*)*-GFP*) and Tg(*FingR*(*GPHN*)*-Cherry*). We drove the FingR constructs using Tg(*otpb.A*:*Gal4*) and then performed double immunohistochemistry for Synapsin and FingR(PSD95)-GFP; or PSD-95 and FingR(PSD95)-GFP; and evaluated for localization of the GFP signal from the FingR neighboring to the pre-synaptic Synapsin, or for co-localization of the GFP signal from the FingR with the post-synaptic PSD-95 (Fig. 2A). To test the fidelity of synapse labeling by FingRs, we counted the number and percentage of synapses showing PSD-95 reactivity alone, GFP alone from FingR(PSD95)-GFP, or co-localization for GFP and PSD-95; in double transgenic larvae Tg(*otpb.A*:*Gal4*); Tg(*FingR*(*PSD95*)*-GFP*). Quantification was performed on sections that had a mixture of neurons and synapses including some that did not express *otpb.A*:*Gal4*, hence not all synapses had FingR expression. We found 95% co-localization (range 91–98%, median 95% +/− 1%, SEM; n = 6 larvae; *p* 0.01 for co-localization) (Fig. 2A inset; Supplementary Data File 1). We validated localization of the FingRs using *ex vivo* preps of zebrafish primary cell culture. We prepared dissociated neurons from Tg(*otpb.A*:*Gal4*); Tg(*FingR*(*PSD95*)*-GFP*) embryos or Tg(*HuC*:*Gal4*); Tg(*FingR*(*GPHN*)*-Cherry*) embryos. When we performed double immunohistochemistry for anti-GFP and anti-PSD95, or anti-mCherry and anti-GPHN, we found that the FingRs localized with the anti-PSD95 or anti-GPHN labeling (Fig. 2B).

A potential concern with use of FingRs in stable transgenic lines would be interference with normal synaptic protein expression, synapse number, or with synapse function. To test whether the FingR system affected expression of synaptic proteins or numbers of synapses, we determined the level of Synapsin expression using immunohistochemistry in defined regions of the telencephalon, or with western blot of the entire embryo, in animals with pan-neuronal expression of FingR(PSD95)-GFP. With pan-neuronal expression of FingRs there were no effects on survival or fecundity. We found no difference in the total number of Synapsin-positive puncta in the telencephalon; and no change in Synapsin expression on westerns (Fig. 2C). To test whether functional properties of neurons were affected by FingR expression we analyzed spontaneous swimming behavior and the audiomotor response in stable transgenic larvae expressing FingRs. When we compared measures of spontaneous as well as of evoked behaviors in larvae with pan-neuronal expression of FingR(PSD95)-GFP we did not find any statistically significant differences (spontaneous behavior: three experimental replicates, n > 20 embryos for each assay; control vs. FingR: movements/second, 0.45 vs 0.40, SEM 0.04 and 0.06, *p* 0.49; percent time swimming, 5.0 vs 5.0, SEM 0.5 and 0.7, *p* 0.82; evoked behavior: three experimental replicates, n = 27 and 30 embryos (control and FingR) velocity (degrees/ms) 20.7 vs 23.0, SEM 2.1 and 3.0, body curvature (degrees) 106 vs 113, SEM 3.3 and 3.7, or latency (ms) 40.7 vs 39.9, SEM 5.4 and 5.0; *p* > 0.5 for all experiments) (Fig. 2D,E). These functional results of motor behavior show that *in vivo* functional properties of synapses were normal even in the presence of pan-neuronal expression of the FingRs.

**Figure 3:**
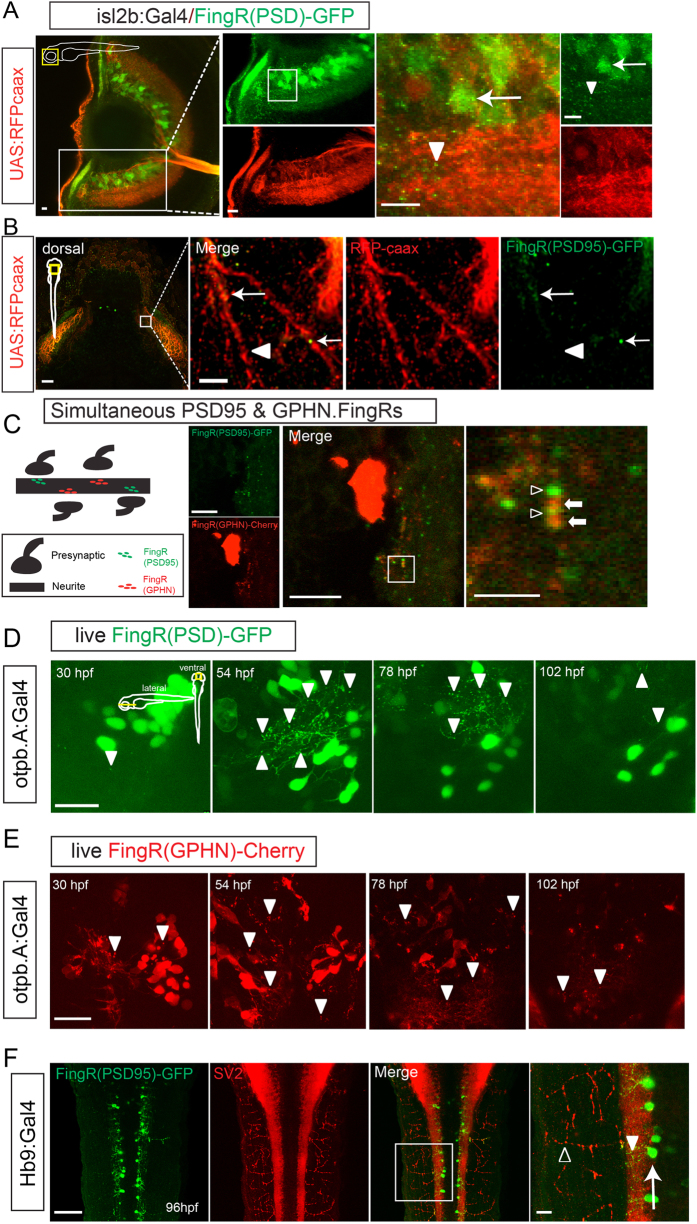
Use of FingRs in visualizing and tracking synapses. (**A**) Lateral view of eye from transgenic zebrafish expressing FingR(PSD95)-GFP and RFP-caax in retinal ganglion neurons (transgenic Tg(*isl2b*:*Gal4*); Tg(*UAS*:*RFP-caax*); Tg(*FingR*(*PSD95*)*-GFP*)). GFP expression is seen in puncta (arrowheads) and neuron somas (arrow); RFP labels neurons, dendrites, and their axons as they project towards optic chiasm (Supplemental Movie 1). Confocal z-stacks at 72 hpf, immunohistochemistry for GFP and RFP, scale bar 10 μm. (**B**) Dorsal view of tectum in Tg(*isl2b*:*Gal4*); Tg(*UAS*:*RFP-caax*); Tg(*FingR*(*PSD95*)*-GFP*) embryo. Arrow shows FingR(PSD95)-GFP puncta along RGC axon, but not along entire length of axon (arrowhead). Confocal z-stack at 72 hpf, immunohistochemistry for GFP and RFP; scale bar 50 μm, 5 μm in inset panel. (**C**) Larvae co-expressing FingR(PDS95)-GFP (open arrowheads) and FingR(GPHN)-Cherry (arrows) demonstrates presence of neighboring excitatory and inhibitory puncta. Confocal z-stacks; scale bar 10 μm, 2.5 μm in inset panel. (**D**,**E**) FingRs can be used for *in vivo* visualization and monitoring of synapses. (**D**) Time-series of dopaminergic neurons and synapses in Tg(*otpb.A*:*Gal4*); Tg(*FingR*(*PSD95*)*-GFP*) animal, demonstrating changes in synapse number and expression with development. Scale bar 20 μm. (**E**) Time-series of dopaminergic neurons and synapses development in Tg(*otpb.A*:*Gal4*); Tg(*FingR*(*GPHN*)*-Cherry*) animal, demonstrating changes in synapse number and expression with development. Scale bar 20 μm. (**F**) Dorsal view in trunk at 96 hpf showing that FingR(PSD95)-GFP is expressed in the spinal cord (arrowhead), but not in axon projections labeled with SV2 (open arrowhead) in the neuromuscular synapses when driven in motor neurons (arrow) by *Hb9*:*Gal4*. Confocal z-stacks at 96 hpf, dorsal views, immunohistochemistry for GFP and SV2; scale bar 50 μm, 10 μm in inset panel.

Next, we tested our transgenic FingR lines for visualization and tracking of synapses in different neuron types and at different ages. With FingR(PSD95)-GFP we could visualize synapses in retinal neurons, and simultaneously detect their axon projections. To do this we used a transgenic line expressing Gal4 in the retinal ganglion cells (RGCs), crossed to a UAS reporter with prenylated RFP for axon visualization, and the regulated UAS FingR (Tg(*isl2b*:*Gal4*); Tg(*UAS*:*RFPcaax*);Tg(*FingR*(*PSD95*)*-GFP*)) GFP expression was seen in the RGC cell bodies and in puncta in the dendritic fields, and RFP expression was seen in the dendrites and axon (Fig. 3A; Supplemental Movie 1). FingR expression did appear limited to the post-synaptic regions: while there was FingR expression along RGC axons^12^, this was consistent with the presence of interneuron synapses onto these axons, and GFP puncta were not present at the termini of the axons or non-specifically along the axons (Fig. 3B). Similarly, in the spinal cord, when expressed in motor neurons, FingR/GFP expression was seen in the neuropil neighboring the neuron cell bodies, but not along the motor neuron axons or near the muscles and neuromuscular junction (Fig. 3F). To test whether we could use FingRs to detect excitatory and inhibitory synapses simultaneously, we visualized FingR(PSD95)-GFP and FingR(GPHN)-Cherry in dopaminergic neurons from the line Tg(*otpb.A*:*Gal4*). We found neurons co-expressing both constructs with multiple distinct puncta of GFP and Cherry (Fig. 3C).

Using the stable transgenic line Tg(*FingR*(*PSD95*)*-GFP*) we could track synapses in live zebrafish, in dopaminergic neurons of the diencephalon. We crossed Tg(*FingR*(*PSD95*)*-GFP*) to Tg(*otpb.A*:*Gal4*), and were able to track changes in synapse number and distribution in live animals (Fig. 3D). After an initial period of exuberant synapse labeling indicated by GFP signal, there was an apparent paring of number and with decreased GFP signal, suggesting relative stability of synapse number and/or PSD-95 protein turnover. We also tracked the changes in distribution and number of puncta with time-lapse confocal movies (Supplemental Movie 2). Similarly, a tracking *in vivo* of GPHN-expressing synapses mirrored the dynamics of PSD-95 (Fig. 3E).

**Figure 4:**
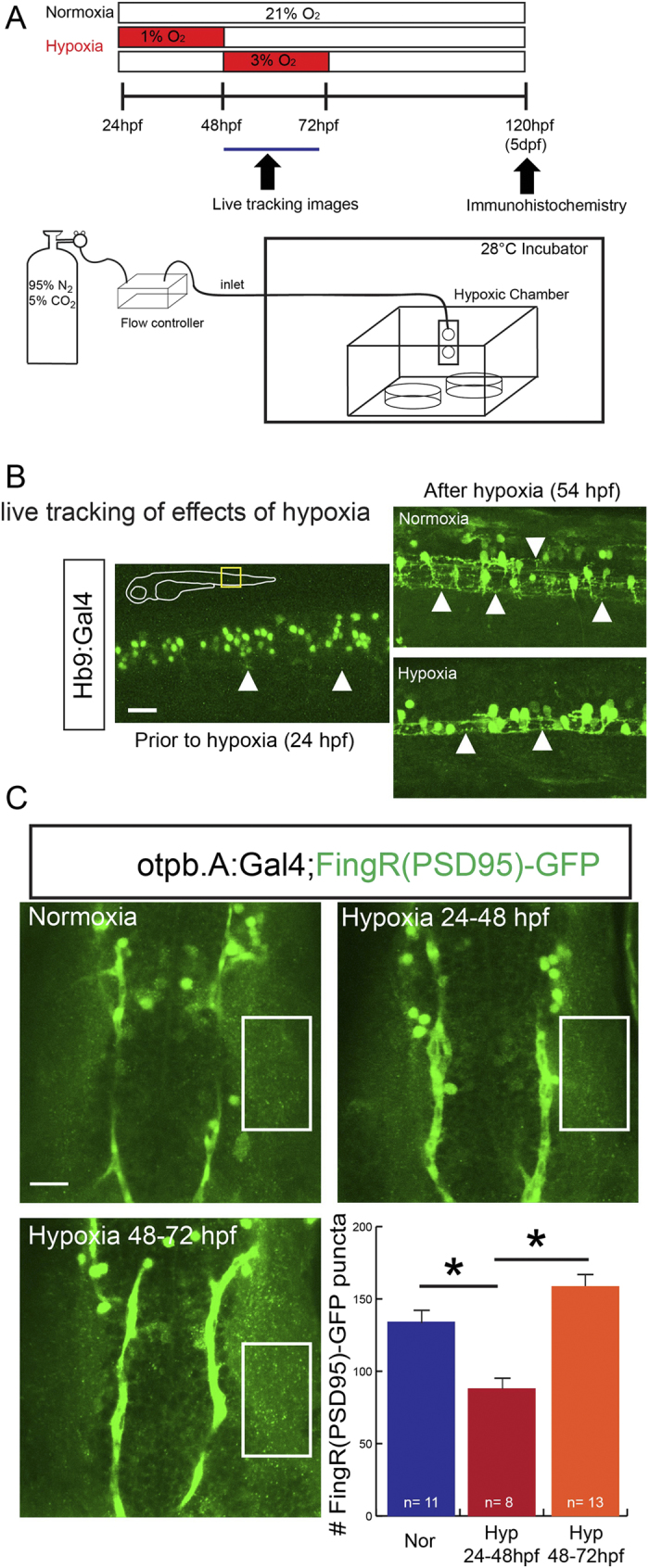
FingRs reveal impairment of synapse development by developmental hypoxic injury. (**A**) Schematic illustration of hypoxia exposure and imaging. (**B**) Demonstration of live imaging of effects of hypoxic injury on motor neuron synaptic puncta visualized by FingR(PSD)-GFP in trunk of Tg(*Hb9*:*Gal4*); Tg(*FingR*(*PSD95*)*-GFP*) animals. Following hypoxia there is a decrease in the number of puncta labeled by FingR(PSD95)-GFP. Confocal images, rostral to left, scale bar 10 μm. (**C**) Confocal images and quantification in the dopaminergic neurons in the diencephalon of Tg(*otpb.A*:*Gal4*); Tg(*FingR*(*PSD95*)*-GFP*) animals. Hypoxia from 24–48 hpf decreased number of puncta but later hypoxia exposure did not significantly change number; (*p* = 0.002; one-way ANOVA; SEM shown). Confocal z-stacks, rostral to top, scale bar 10 μm.

Hypoxic injury complicates up to 60% of preterm births and leads to a broad range of neurological birth defects including epilepsy, autism, ADHD, and mental retardation^13^. Despite the significant clinical impact, the specific effects on the developing central nervous system (CNS) following hypoxia are poorly characterized. We had shown previously that hypoxia can specifically disrupt axon guidance without causing an increase in CNS apoptosis^14^, but the effects of hypoxia on synapse generation and identity in different neuron populations is unknown. To study this we used a model of chronic developmental hypoxia (Fig. 4A)^14^. With FingRs we found that we could track in live animals the effects of hypoxia on synapse maturation in motor neurons of the spinal cord (Fig. 4B). Following hypoxia there was a decrease in the number of puncta associated with spinal cord motor neurons in live animals. We were particularly interested in the effects of hypoxia on dopaminergic neuron populations of the diencephalon, because of their roles in essential aspects of behavior and clinical relevance in the potential pathogenesis of motor disorders in premature birth^15^. We found that developmental hypoxia had differing effects on the number of dopamine neuron synapses depending on the timing of hypoxic exposure (Fig. 4C). An early exposure to hypoxia (24 to 48 hpf) led to a decrease in FingR(PSD95)-GFP puncta (85.9 +/− 7.3); in contrast, later hypoxia (48 to 72 hpf) was not associated with a change in puncta number (hypoxia 158.6 +/− 12.5 vs normoxia 134.1 +/− 11.6) (one-way ANOVA SEM; *p* = 0.002 24–48 hpf compared to 48–72 hpf; *p* = 0.029 24–48 hpf compared to normoxia).

## Discussion

Our implementation of an inducible expression system for FingRs in transgenic zebrafish provides a novel approach for visualizing and quantifying development, dynamics, and the effects of experimental or disease manipulations on the post-synaptic proteins PSD-95 and GPHN. We have generated stable transgenic lines with FingRs that bind and label the endogenous PSD-95 and GPHN, but with a negative feedback mechanism to limit expression levels of the FingRs. In control experiments we found no changes in synapse structure or in behavior in animals expressing FingRs. Our approach is built upon an inducible expression system using Gal4/UAS; and we have shown that *in vivo* monitoring of synapse maturation and dynamics is feasible.

We used our transgenic FingR system to demonstrate their utility for studying the effects of developmental hypoxia on synapse structure. While developmental hypoxia is known to alter expression of synaptic genes^16^,^17^ the actual effects on number and distribution of synapses in different neurons has not been characterized. Using FingRs we were able to observe alterations in the number of excitatory synapses of dopaminergic neurons. Dopaminergic neurons are important for regulation of motor tone and movement and are injured in infants with cerebral palsy and prematurity^15^. Interestingly, the effect differed dependent on the developmental timing of hypoxic exposure, with early hypoxia associated with a decrease in synapse number; while later hypoxia did not change synapse number. This provides insights into the neuropathophysiology and molecular mechanisms associated with the effects of prematurity on brain development. Also, this reveals that hypoxia is not a single insult, but that its effects are linked to developmental changes in the CNS, which has implications for treatment strategies.

In summary, our results demonstrate that FingR technology can be successfully applied in transgenic zebrafish for expression in genetically distinct neuron subpopulations with the ability to differentially visualize inhibitory or excitatory synapses. FingRs can be used for tracking of live synapse dynamics and in analysis of effects in defined neuron types.

## Methods

### Ethics Statement and Fish Care

Zebrafish experiments were performed in accordance with guidelines, and approved by the University of Utah Institutional Animal Care and Use Committee (IACUC), protocol 14-04017, approved 04/21/2015, regulated under federal law (the Animal Welfare Act and Public Health Services Regulation Act) by the U.S. Department of Agriculture (USDA) and the Office of Laboratory Animal Welfare at the NIH, and accredited by the Association for Assessment and Accreditation of Laboratory Care International (AAALAC). Breeding and embryo care was performed according to standard methods^18^.

### Fish stocks and transgenic fish lines

Transgenic fish lines and alleles used in this paper were the following: Tg(*otpb.A*:*Gal4-VP16*_*413*−*470*_; *myl7*:*EGFP*)^zc57^ (referred to as Tg(*otpb.A*:*Gal4*))^11^; Tg(*isl2b.3*:*Gal40VP16*_*413*−*470*_; *myl7*:*TagRFP*)^zc65^ (referred to as Tg(*isl2b*:*Gal4*))^19^; Tg(*foxP2.A.2*:*Gal4-VP16*_*413*−*470*_; *myl7*:*EGFP*)^zc72^ (referred to as Tg(*foxP2.A.2*:*Gal4*)^14^; and Tg(*elavl3*:*Gal4-VP16*_*413*−*470*_)^zc87^ (referred to as Tg(*Huc*:*Gal4*))^14^. Injection of DNA constructs and generation of stable transgenic lines was performed as described^20^. New lines generated were Tg(*zcUAS*:*PSD95.FingR-GFP-ZFC*(*CCR5TC*)*-KRAB*(*A*))^zc88^ and Tg(*ziUAS*:*GPHN.FingR-mCherry-ZFI*(*IL2RGTC*)*-KRAB*(*A*))^zc89^. See Supplemental Table 2. Lines are available at Zebrafish International Resource Center (ZIRC) (Eugene, OR) or upon request.

### Cloning

Cloning for use in zebrafish was based on the Tol2 kit and recombination reactions with Gateway (Invitrogen) plasmids^21^. Identity of constructs was confirmed by restriction enzyme digests and by sequencing of both strands (for coding sequences). Plasmids used for cloning were pDONR221; p5E-10xUAS; pME-mCherry; p3E-pA; and pDestTol2pA2^21^. Plasmids pCAG_PSD95.FingR-eGFP-CCR5TC-KRAB(A) and pCAG_GPHN.FingR-mKate2-IL2RGTC-KRAB(A) were obtained from Addgene (plasmids #46295 and #46297). The PSD95.FingR-eGFP-CCR5TC-KRAB(A) open-reading frame was PCR amplified and BP cloned into pDONR221 to generate pME-PSD95.FingR-eGFP-CCR5TC-KRAB(A) (primers were forward, 5′-GGGGACAAGTTTGTACAAAAAAGCAGGCTACCATGCTCGAAGTCAAGGAAGCATCA-3′, and reverse 5′-GGGGACCACTTTGTACAAGAAAGCTGGGTATTAAGCCATAGAAGCAAGAT-3′). To generate p5E-zcUAS, p5E-10xUAS was digested with KpnI and HindIII; a PCR amplicon with KpnI/HindIII compatible sites containing the GTCATCCTCATC sequence (the CCR5TC zinc finger domain binding site) (primers were forward, 5′-TATAGGGCGAATTGGGTACCAAGGGAATAAGGGCGACACG-3′; and reverse: 5′-TTGGTGGCCTAAGCTTACCGTAAATAGTCCACCCATTG-3′) was ligated into the linearized p5E-10xUAS. pTol2-zcUAS:PSD95.FingR-eGFP-CCR5TC-KRAB(A) was generated by an LR reaction. To generate pME-UAS:GPHN.FingR-mCherry-IL2RGTC-KRAB(A), we first generated the pME clone GPHN.FingR-mKate2-IL2RGTC-KRAB(A) in pDONR221 using the same primers as for the PSD95.FingR PCR amplification. We replaced mKate2 with mCherry by cutting pME-GPHN.FingR-mKate2-IL2RGTC-KRAB(A) with BamHI and MscI, then mCherry was ligated in using pME-mCherry as a template for PCR amplification with primers containing BamHI and MscI sites (forward, 5′-ATCAACTACCGCACCGGATCCATGGTGAGCAAGGGC-3′, reverse, 5′-GGAGGTCGCAGTATCTGGCCACTTGTACAGCTCGTCC-3′).

To generate p5E-ziUAS, we cloned the zinc finger binding site for IL2RGTC upstream of UAS. We linearized p5E-10XUAS with KpnI and HindIII, and ligated in a KpnI/HindIII PCR fragment amplified from pCAG_GPHN.FingR-mKate2-IL2RGTC-KRAB(A) containing the IL2RGTC zinc finger binding sites (5′-CTTCCACAGAGT-3′) (primers forward, 5′-TATAGGGCGAATTGGGTACCAAGGGCGACACGGAAATGTTG-3′; and reverse, 5′-TGGAGGCCTAAGCTTACCGTAAATAGTCCACCCATTG-3′). pTol2-ziUAS:GPHN.FingR-mCherry-ZFI(IL2RGTC)-KRAB(A) was generated by a LR reaction. Schematic diagrams shown in Fig. 1A; list at Supplemental Data Table 2.

### Immunohistochemistry and double immunohistochemistry

Immunohistochemistry was performed as previously described^20^. Antibodies used were: mouse monoclonal anti-GFP 1:250 (Millipore), rabbit anti-synapsin 1:1000 (Synaptic Systems)^22^, rabbit anti-PSD95 (Abcam)^23^, Cy-3 anti-rabbit 1:400, Alexa 488 donkey anti-mouse 1:400 (Invitrogen), and rabbit anti-goat Alexa 555 (Invitrogen). Double immunohistochemistry for GFP with anti-synapsin or anti-PSD95 was performed at 3 dpf as follows: embryos were fixed in 4% paraformaldehyde (PFA) in PBS for 1.5 hours at room temperature (RT) and washed briefly in PBS (3 × 5 min) with 0.1% Triton X-100 (PBST). Embryos were then blocked (PBS with 1% BSA, 1% DMSO, 2% goat serum, and 0.1% Triton X-100) for 3 hours at RT, and then incubated in blocking buffer with primary antibodies overnight at 4 °C. Embryos were washed with PBST, and incubated with secondary antibodies overnight at 4 °C.

For sectioning, stained larvae was cryoprotected in 30% of sucrose with PBS for at least 2 hr, rinsed briefly in Tissue-Tek O.C.T. compound (Sakura Finetek, Torrance, CA), and frozen in a Tissue-Tek O.C. ethanol/dry ice bath. Sections were 20 μm and mounted on the Superfrost Plus Microscope slides.

### Western Blot

Fifty larvae (3 dpf) were deyolked by triturating and incubating in deyolking buffer (65 mM NaCl, 1.7 mM KCl, and 1.5 mM NaHCO_3_) for 10 min at RT. Protein was extracted by grinding larval fish with pestle in a 100 μl of lysis buffer (150 mM NaCl, 20 mM Tris-HCl, pH7.5, 1 mM EDTA, 1% NP-40, and 1% Triton X-100, 1x Halt Protease and Phosphatase Inhibitor Cocktail, (Life Technologies, Grand Island, NY)) on ice. The extract was centrifuged for 10 min (16,000 g RCF) at 4 °C and the supernatant was transferred to a new tube. Total protein concentration was determined by BCA assay (Thermo Scientific Pierce BCA Protein Assay kit, Fisher Scientific, Pittsburgh, PA). The protein extract was then mixed with an equal amount of 2x Laemmli buffer (20% glycerol, 4% SDS, 0.1% bromophenol blue, 0.125 M Tris pH 6.8, 2.5% β-mercaptoethanol) and boiled for 3 min. Samples were stored at −20 °C until use. 20 μg of total protein was loaded on to each lane of a 4–20% polyacrylamide gel (Mini-Protean TGX Gel Bio-rad, Hercules, CA). After electrophoresis proteins were transferred to PVDF membranes and blocked in 3% non-fat dry milk in TBS (50 mM Tris, 0.138 M NaCl, 2.7 mM KCl, pH 8.0) for 30 min at RT with agitation. Membranes were split, and then incubated in rabbit anti-synapsin (1:1000) or rabbit anti-β-catenin (1:1000) for 2 hr at RT; washed in TBS; and then incubated in HRP-anti-rabbit (1:5000 dilution) for 1 hr at RT. Following incubation with secondary antibody, membranes were washed extensively in TBS containing 0.05% Tween-20, and then subjected to chemiluminenscent detection (Clarity Western ECL Substrate (Bio-Rad, Hercules, CA). The western blot, imaging and quantification was performed with a digital imager (Gel Do XR + System and Image Lab Software, Bio-Rad, Hercules, CA) in three separate experimental replicates.

### Cell culture

#### Isolation of zebrafish primary neurons

Embryos expressing FingRs were incubated at 28.5 °C until 24–48 hpf; ~100 embryos were used to initiate each primary cell culture. Embryos were rinsed in phosphate-buffered saline (PBS) and treated with 0.125% bleach solution (NaOCl) for 5 minutes at room temperature (RT). Embryos were then treated with 2 mg/ml Pronase at 28.5 °C for 20 minutes and rinsed with PBS. The dechorionated embryos were exposed to 0.05% Trypsin/EDTA (Life Technologies) for 5 minutes at RT, and fetal bovine serum (FBS) was added to terminate trypsinization. 24–48 hpf embryos remain intact during this step. Embryos were digested in 1 mg/ml collagenase while rocking at RT for 15 minutes. Using a 1000 μl pipette tip, embryos were triturated and incubated for 5 additional minutes at RT. The digested cells were then filtered through a 70-μm filter and centrifuged at 600 xg (rcf) for 8 minutes. Pelleted cells were re-suspended in 1 ml zebrafish cell culture media and filtered through a 40-μm filter and centrifuged as in the previous step. Zebrafish cell culture media consisted of 50% Leibowit’s L-15 (Invitrogen), 30% high-glucose Dulbecco’s modified Eagle’s medium (DMEM; Invitrogen), 15% Ham’s F-12 (Invitrogen), 0.18 g/l sodium bicarbonate (Sigma), 15 mM HEPES (Invitrogen), 100 μM Penicillin/Streptomycin/L-glutamine (Invitrogen), 10 μg/ml Ciprofloxacin (Sigma), 10 nM sodium selenite (Sigma), 1x N-2 supplement (Invitrogen), 1x B-27 serum free supplement (Invitrogen), 10 ng/ml Nerve Growth factor 7S (Invitrogen), 15% fetal bovine serum (Invitrogen), 1% rainbow trout serum (Caisson Labs). The pelleted cells were then re-suspended in 1 ml zebrafish cell culture media. To each well of a 6-well plate, 150 μl of re-suspended cells was added to 3 ml of zebrafish cell culture media. Each well contained a cover slip (VWR 24 × 24 mm, No. 0) treated with 0.01% poly-L-lysine solution (Sigma), and 10 μg/ml natural mouse laminin (Invitrogen). Cultures were incubated at 28.5 °C without supplemental CO_2_. For each day in cell culture, 1 ml of media was removed and replaced with fresh media.

### Cell culture immunocytochemistry

At 3–5 days post *in vitro* (dpiv), cells that had adhered to the coverslips were washed several times in PBS to remove media and fixed in 4% para-formaldehyde (PFA) for 5 minutes at RT. Cells on coverslips were then washed several times in PBS, and then incubated in blocking solution containing 2% bovine serum albumin (Amresco), and 5% normal goat serum (Sigma) in PBS, for 1.5 hours. Cells were then incubated in primary antibody (mouse anti-GFP, 1:250, EMD Millipore; rabbit anti-PSD95, 1:1000, Abcam; mouse anti-GPHN, 1:1000, Synaptic Systems; rabbit anti-dsRed 1:250,Clontech), diluted in blocking solution, for 1.5 hours at RT. Cells were washed several times in PBS to remove primary antibody. Cells were subsequently incubated in secondary antibody (Goat anti-mouse IgG Superclonal secondary, Alexa588, 1:400, Thermo Scientific; Goat anti-Rabbit IgG, Cy3 conjugate, 1:400, EMD Millipore) with DAPI (1:100; 4′,6-diamidino-2-phenylindole; Invitrogen) diluted in blocking solution and incubated for 30 minutes at RT. Cells were washed in PBS and post-fixed in 4% PFA for 1 minute. Coverslips were mounted on super-frost slides (VWR) in 80% glycerol for imaging.

### Startle response kinematics and free-swimming behavioral analyses

Larval fish 6–8 days post-fertilization (dpf) were immobilized in 1.5% low-melt agarose and mounted in a 3.5 cm dish flooded with E3 solution. The tail was subsequently freed from the agarose to allow tracking of tail movements during startle responses. Fish were allowed to habituate to the agarose on the imaging platform for 10 minutes prior to experimentation. Fish were imaged with a Pike IEEE 1394b camera (Allied Vision Technologies) at 544 frames per second. Startle stimuli consisted of a 1 KHz acoustic/vibrational pulse delivered by a speaker mounted directly to the imaging platform 6 cm away from the dish. Image acquisition and stimulus delivery were driven using custom software written in LabView (National Instruments).

Tail tracking was accomplished using custom software written using MatLab (Mathworks, Inc.). The software generated a binary image of the fish using a user-defined threshold, with a series of morphological operations to define six tracking points at equal distances along the body axis of the fish. Total tail curvature in each frame was calculated as the sum of the angles between each of the tracking points, and angular velocity was derived from this value. Latency was defined as the time to initiation of movement after stimulus presentation.

7 dpf larval fish were placed singly in a circular behavior arena 3.5 cm in diameter and 7 mm high filled with E3 embryo water and given 10 minutes to habituate to the arena. Trials consisted of 10 minutes of undisturbed observation per fish. Spontaneous motor behaviors were counted using a running frame subtraction algorithm in which spikes in pixel intensity difference corresponded to movement of the fish. Spikes were located using custom MatLab software. Percent time spent swimming was calculated as percentage of frames in which the total pixel intensity difference was above threshold. Fish were imaged at 60 frames per second. Image acquisition and running frame subtraction were driven using custom LabView software.

### Microscopy and image analysis

Image acquisition and analysis were performed as described previously^20^. Immunostained embryos were transferred step-wise into 80%glycerol/20% PBST, mounted on a glass slide with a #0 coverslip placed over a well made using electrical tape, and imaged on a confocal microscope. Confocal stacks were projected in ImageJ, and images composed with Adobe Photoshop and Illustrator. For the live tracking of FingRs, larval fish were immobilized in a drop of 1% low-melt agarose and mounted to a 3.5 cm dish flooded with E3 embryo water for 1 hr in each day from 2 dpf to 6 dpf.

### Quantification of Synapsin and zcPfingR-GFP Puncta counts

Tg(*elavl3*:*Gal4-VP16*_*413*−*470*_); Tg(*zcUAS*:*PSD95.FingR-GFP-ZFC*(*CCR5TC*)*-KRAB*(*A*)), (n = 9) and Tg(*elavl3*:*Gal4-VP16*_*413*−*470*_) (n = 9) larvae (3 dpf) were used to analyze the number of synaptic puncta stained by Synapsin. We imaged serial confocal sections at 1.14 μm intervals over a depth of 63 μm for a total of 55 optical sections. 3 sections (1^th^, 5^th^, and 10^th^ section) from each animal were used for counts; the ventral-most section where the anterior commissure was first imaged was section #1. Puncta were counted from a circle of set size (W: 30.0 μm × H: 30.0 μm) placed in the diencephalon on the longitudinal axon tract in section #1, immediately medial to the edge of the eye and at the rostral edge where the longitudinal tract was first apparent. The total number of puncta in the circle was counted from three sections/animal and averaged. The background of each image was subtracted using a 5 pixel radius Rolling Ball function (ImageJ, “Subtract Background”), using auto threshold. The number of puncta was counted using “Analyze Particles” (ImageJ). For the zcPfingR-GFP puncta counting between normoxic and hypoxic larvae (5 dpf), puncta were counted in a rectangle of set size (W: 30.0 μm × H: 50.0 μm) placed in the diencephalon with its medial edge on the longitudinal axon tract, and its rostral/caudal location at the midline of the lens. The total number of puncta in the rectangle was counted from 10 sections (1.0 μm thickness) /animal and averaged. Each image was set for the threshold between (45–255) and adjusted by binary function (Watershed). Size for puncta was set as 0.5–20 μm^2^. The number of puncta was counted using “Analyze Particles” (ImageJ).

### 3-D Image and Movie Analysis

For 3-D images, confocal images were processed with FluoRender^24^. Rendered images were exported as TIFFs and converted into avi files using ImageJ.

### Hypoxia

For induction and monitoring of hypoxia, embryonic zebrafish were placed in a sealed plexiglass chamber^14^. Embryos were incubated in 1% O_2_ from 24–48 hpf.

### Statistical analysis

Statistical analysis was performed using Student’s t-test, or one-way ANOVA (treatment) and Tukey-Kramer HSD post-hoc test; with a significant *p* value set as *p* ≤0.05.

## Additional Information

**How to cite this article**: Son, J.-H. *et al.* Transgenic FingRs for Live Mapping of Synaptic Dynamics in Genetically-Defined Neurons. *Sci. Rep.* **6**, 18734; doi: 10.1038/srep18734 (2016).

## Supplementary Material

Supplementary Information

Supplemental Data Table 1

Supplemental Movie 1

Supplemental Movie 2
