# Supplementary material for: Transgenic FingRs for Live Mapping of Synaptic Dynamics in Genetically-Defined Neurons
Source: Sci Rep. 2016 Jan 5;6:18734. doi: 10.1038/srep18734 (PMC4700522; doi:10.1038/srep18734)
Supplement: Supplementary Information [file srep18734-s1.docx]

**Supplemental Information**

**Transgenic FingRs for Live Mapping of Synaptic Dynamics in Genetically-Defined Neurons**

Jong-Hyun Son, Tamara J. Stevenson, Joshua P. Barrios, Scott Anjewierden, James B. Newton, Adam D. Douglass, and Joshua L. Bonkowsky

**Supplemental Data File 1**. Co-localization of rFingR-GFP and PSD-95. Data is from six planes in the telencephalon (n=6 animals) of Tg(*otpb.A:Gal4*); Tg(*FingR(PSD95)-GFP*) embryos immunostained and sectioned.

**Supplemental Table 2**. List of FingR-related constructs and transgenic fish lines generated; ORF, open reading frame.

**Constructs**

Name and Description

*pME-PSD95.FingR-EGFP*

ORF for PSD95.FingR-EGFP

*pME-PSD95.FingR-EGFP-CCR5TC-KRAB(A)*

ORF for PSD95.FingR-EGFP with zinc finger domain CCR5TC and KRAB(A) inhibitor

*pME-GPHN.FingR-mKate2-IL2RGTC-KRAB(A)*

ORF for GPHN.FingR-mKate2 with zinc finger domain IL2RGTC and KRAB(A) inhibitor

*pME-GPHN.FingR-mCherry-IL2RGTC-KRAB(A)*

ORF for GPHN.FingR-mCherry with zinc finger IL2RGTC and KRAB(A) inhibitor

*p5E-ziUAS*

binding site for zinc finger domain IL2RGTC cloned upstream of UAS

*p5E-zcUAS*

binding site for zinc finger domain CCR5TC cloned upstream of UAS

*pTol2-zcUAS:PSD95.FingR-EGFP-CCR5TC-KRAB(A)*

final Tol2 transgene plasmid with PSD95.FingR; EGFP; CCR5TC domain; and KRAB(A) domain; and binding site for zinc finger domain CCR5TC cloned upstream of UAS

*pTol2-ziUAS: GPHN.FingR-mCherry-IL2RGTC-KRAB(A)*

final Tol2 transgene plasmid with GPHN.FingR; mCherry; IL2RGTC domain; and KRAB(A) domain; and binding site for zinc finger domain IL2RGTC cloned upstream of UAS

**Transgenic Fish Lines**

Name Allele

Tg(*zcUAS:PSD95.FingR-GFP-ZFC(CCR5TC)-KRAB(A*)) zc88

Tg(*ziUAS:GPHN.FingR-mCherry-ZFI(IL2RGTC)-KRAB(A))* zc89

**Supplemental Movie 1.** Lateral view movie of eye from transgenic zebrafish Tg(*isl2b:Gal4*); Tg(*UAS:RFP-caax*); Tg(*FingR(PSD95)-GFP*) expressing FingR(PSD)-GFP and RFP-caax in retinal ganglion neurons. GFP expression is seen in neuron somas and puncta; RFP labels neurons, dendrites, and their axons are they project towards optic chiasm.

**Supplemental Movie 2.** Coronal view (dorsal to left) time-lapse movie of olfactory neurons in live transgenic zebrafish Tg(*otpb.A:Gal4*); Tg(*FingR(PSD95)-GFP*). GFP expression is seen in olfactory neurons cell bodies, and in puncta in dendrites extending from these neurons.
